# CircRNA FUT10 regulates the regenerative potential of aged skeletal muscle stem cells by targeting HOXA9

**DOI:** 10.18632/aging.203233

**Published:** 2021-07-13

**Authors:** Menghai Zhu, Chong Lian, Gang Chen, Peng Zou, Beng Gang Qin

**Affiliations:** 1 Department of Foot and Ankle Surgery, Center for Orthopaedic Surgery, The Third Affiliated Hospital of Southern Medical University, Guangzhou 510630, Guangdong, PR. China; 2 Department of Orthopedic, The First Affiliated Hospital, Sun Yat-Sen University, Guangzhou 510080, Guangdong, PR. China

**Keywords:** aging, circRNA FUT10, HOXA9, skeletal muscle stem cells

## Abstract

Skeletal muscle is capable of repairing itself after injury to maintain the stability of its own tissue, but this ability declines with aging. Circular RNAs (circRNAs) are involved in cell aging. However, there is little research into their role and underlying mechanisms, especially in skeletal muscle stem cells (SkMSCs). In this study, we assessed circRNA FUT10 expression in aged and adult SkMSCs. We observed that circRNA FUT10 was upregulated in aged SkMSCs compared with that in adult SkMSCs. Furthermore, we identified putative miR-365-3p binding sites on circRNA FUT10, suggesting that this circRNA sponges miR-365a-3p. We also found that HOXA9 is a downstream target of miR-365a-3p and confirmed that miR-365a-3p can bind to circRNA FUT10 and the 3′-untranslated region of HOXA9 mRNA. This finding indicated that miR-365a-3p might serve as a “bridge” between circRNA FUT10 and HOXA9. Finally, we found that the circRNA FUT10/miR365a-3p/HOXA9 axis is involved in SkMSC aging. Collectively, our results show that the circRNA FUT10/miR365a-3p/HOXA9 axis is a promising therapeutic target and are expected to facilitate the development of therapeutic strategies to improve the prognosis of degenerative muscle disease.

## INTRODUCTION

Age-related factors and progressive homeostatic decline are among the most common health issues for individuals at advanced age disease, such as amyotrophic lateral sclerosis (ALS), Duchenne muscular dystrophy (DMD) and sarcopenia, et al [1, 2]. Degenerative muscle disease, attributable to skeletal muscle aging, is a common age-related disorder. Skeletal muscle can repair itself after injury. This process is largely dependent on skeletal muscle stem cells (SkMSCs), and their repairability declines with aging [3, 4]. Therefore, exploring the mechanisms underlying skeletal muscle aging is essential for the clinical treatment of degenerative muscle disease.

Circular RNAs (circRNAs) are a class of single-stranded noncoding RNA molecules, that are involved in age-related diseases [5–8]. Panda et al. reported that the senescence-associated circular RNA (SAC-RNA) circPVT1 is markedly reduced in senescent fibroblasts, promotes cell senescence and reverses cell proliferation by selectively targeting let-7 [9]. Moreover, Liang et al. reported that in lens epithelial cells, circRNA ZNF292 plays a role in resistance to oxidative damage by modulating miR-23b-3p in age-related cataracts (ARCs) [10]. Notably, recent studies suggest that circFUT10 promotes proliferation and inhibits differentiation via sponging let-7 [11]. In addition, circRNA FUT10 regulates myoblast cell survival and aging [12]. However, the role and mechanism underlying circRNA FUT10 activity in SkMSC aging is sparsely reported.

CircRNAs usually exert their biological effect s by binding to microRNAs (miRNAs) and modulating their normal function in cells. Zhao et al. reported that the circ RNA circ_0072995 promotes epithelial ovarian cancer (EOC) progression [13]. Similarly, many studies have reported the relationship between miRNAs and circRNAs in SkMSCs [14–16]. Interestingly, complementary sites between miR-365a-3p and circRNA FUT10 were predicted by bioinformatics analysis. Previous works demonstrated that miR-365a-3p is involved in the progression of colorectal cancer [17], cerebral infarction [18], small and large abdominal aortic aneurysms [19], gastric cancer [20], myeloid leukemia [21], and laryngeal squamous cell carcinoma [22]. However, the mechanism involved in circRNAFUT10 and miR-365a-3p interactions needs to be further studied. In addition, Homeobox protein Hox-A9 (HOXA9) is related to denervated muscle atrophy [23], and inhibiting HOXA9 improved SkMSCs function and muscle regeneration in aged mice [24]. Similar to circRNA FUT10, bioinformatic analysis predicted the presence of binding sites for miR-365a-3p in the 3′-untranslated regions (UTRs) of HOXA9 mRNA. Based on these findings, we hypothesized that circRNA FUT10 regulates HOXA9 by sponging miR-365a-3p.

In this study, we examined circRNA FUT10, miR-365-3p, and HOXA9 expression in adult and aged SkMSCs. We also investigated the biological role of these molecules in aged SkMSCs. Finally, we determined whether the circRNA FUT10/miR-365a-3p/HOXA9 axis is involved in regulating SkMSCs aging. Our results will contribute to our understanding of degenerative muscular disease and identify therapeutic targets to improve disease prognosis.

## RESULTS

### Analysis of differentially expressed circRNAs between adult and aged SkMSCs

**Figure 1:**
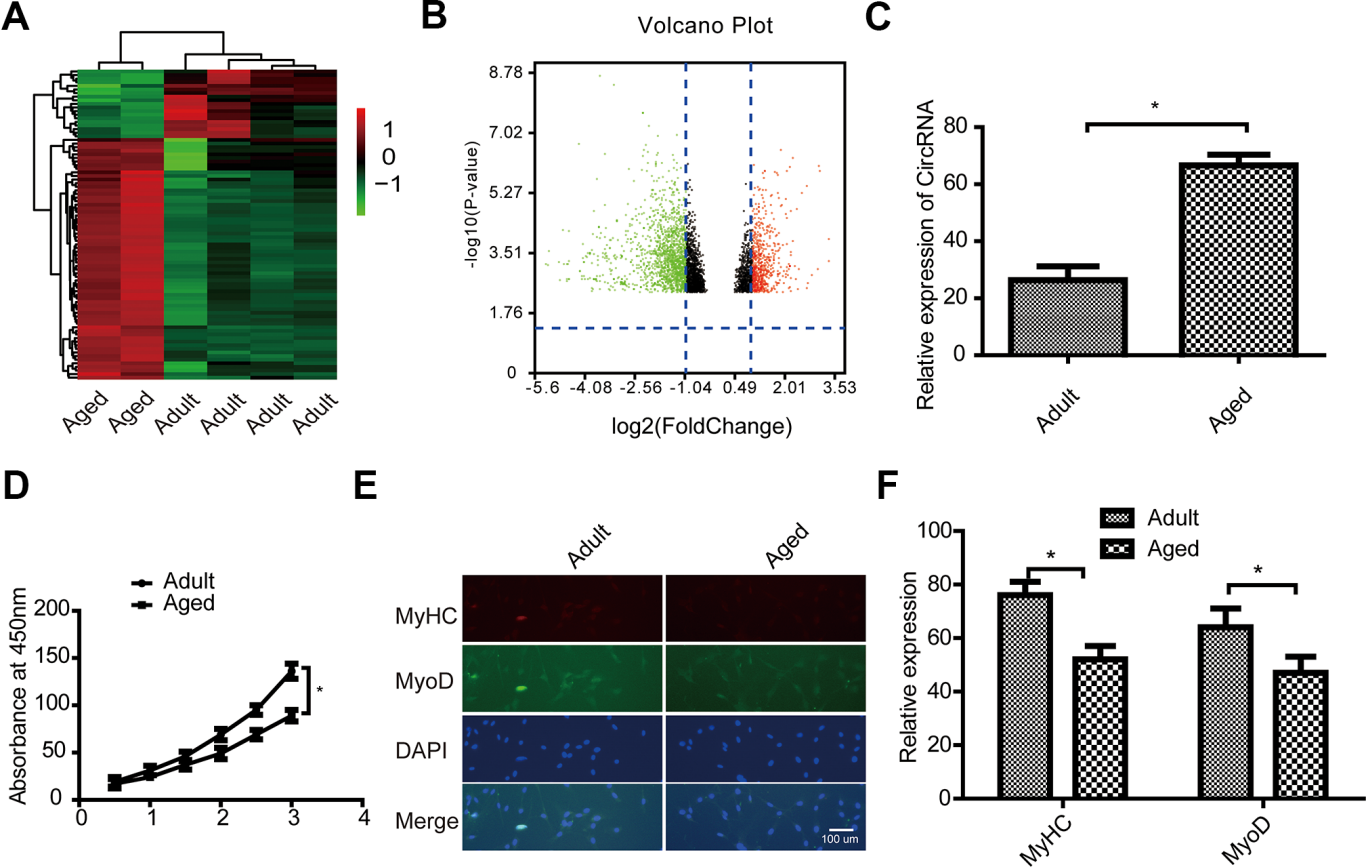
**Characterization of circular RNA (circRNA) expression profiles in adult and aged SkMSCs.** (**A**) Volcano plots were used to evaluate differences in circRNA expression between adult and aged SkMSCs. The horizontal line represents a 2.0-fold (log2 scaled) difference in expression and the vertical lines represent P = 0.05 (–log10 scaled). (**B**) Hierarchical clustering of circRNA expression profiles. Red and green indicate high and low relative expression, respectively. The red and green points in the plot represent the significantly differentially expressed circRNAs. (**C**) circRNA FUT10 mRNA expression in the above two groups. (**D**) MTT assay showing adult and aged SkMSC proliferation. (**E**) Immunofluorescence analysis of MyHC and MyoD expression in adult and aged SkMSCs. (**F**) Quantification of MyHC and MyoD. Each bar represents the mean ± SEM. *P < 0.05, **P < 0.01. All experiments were performed at least three times with duplication within each individual experiment.

To further explore the potential functions of circRNA FUT10, we prepared a clustered heatmap. In total, 9,572 expressed circRNAs were detected using an Arraystar mouse circRNA microarray. We then identified circRNAs that were differentially expressed between adult and aged SkMSCs. Hierarchical clustering was performed to sort circRNAs according to their expression level (Figure 1A). In the volcano plots (Figure 1B), differentially expressed circRNAs were grouped by fold change (FC) ≥ 2.0, P < 0.05, and false discovery rate < 0.05. This analysis 264 significantly different circRNAs in aged SkMSCs compared with adult SkMSCs. Of these, 152 circRNAs were upregulated, and 112 were downregulated. We selected circRNA FUT10, one of the most upregulated circRNAs and then verified it by qRT-PCR (Figure 1D). MTT cell proliferation assays showed that adult SkMSCs have higher proliferative capacity than aged SkMSCs. In addition, MyoD and MyHC expression was measured in adult and aged SkMSCs. Immunofluorescence analysis indicated that adult SkMSCs show higher differentiation ability (Figure 1E, 1F) than aged SkMSCs. Together, these data indicate that circRNA FUT10 is differentially expressed in adult and aged SkMSCs and plays a role in SkMSC proliferation and differentiation.

### Effect of circRNA FUT10 on SkMSCs proliferation and differentiation

**Figure 2:**
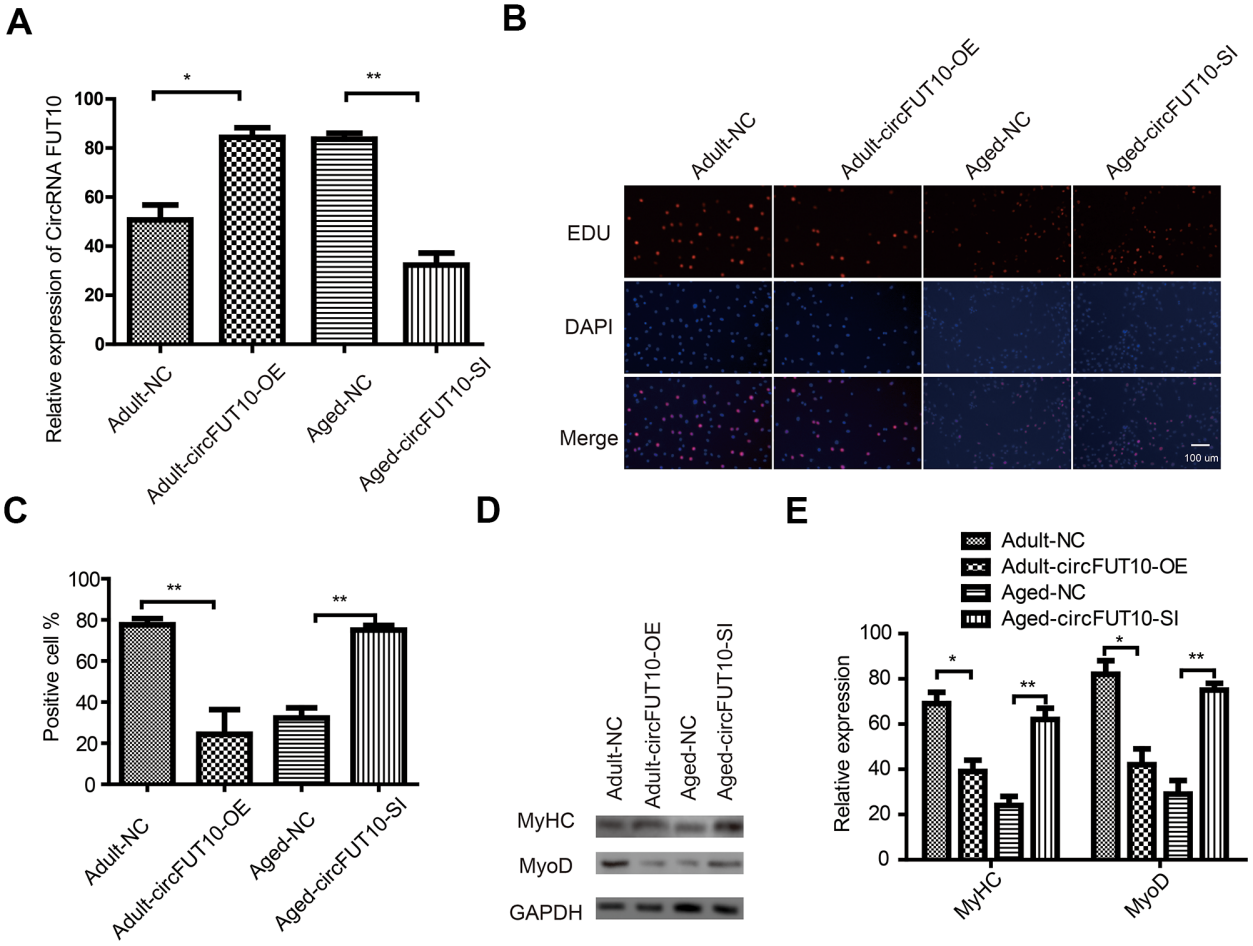
**Effect of circFUT10 on SkMSC proliferation and differentiation.** (**A**) Adult SkMSCs were transfected with circRNA FUT10 overexpression vector, while aged SkMSCs were transfected with circFUT10 small interfering RNA for circRNA FUT10 knock down. circRNA FUT10 mRNA was analyzed using real-time qPCR. (**B**) SkMSC proliferation was measured by EdU assays. (**C**) Quantification of EdU-positive cells. (**D**) Western blot analysis showing MyHC and MyoD expression in different groups. (**E**) Quantification of MyHC and MyoD were normalized to β-actin. Each bar represents the mean ± SEM. *P < 0.05, **P < 0.01. All experiments were performed at least three times with duplication within each individual experiment.

To further explore the potential functions of circRNA FUT10 in adult and aged SkMSCs, we designed circRNA FUT10 overexpression vectors and short hairpin RNA (shRNA) to knock down circRNA FUT10 (Figure 2A). These constructs were transfected into SkMSCs. EdU assays showed that circRNA FUT10overexpression inhibited adult SkMSC proliferation, while suppressing circRNA FUT10 with shRNA promoted aged SkMSC proliferation (Figure 2B, 2C). Next, the effect of circRNA FUT10 on differentiation was evaluated by western blot of the differentiation markers MyoD and MyHC. circRNA FUT10 overexpression reduced MyoD and MyHC expression and inhibited the differentiation of adult SkMSCs, while circRNA FUT10 suppression promoted the differentiation of aged SkMSCs (Figure 2D, 2E). These data indicate that circRNA FUT10 regulates proliferation and differentiation in adult and aged SkMSC.

### circRNA FUT10 suppresses the proliferation and differentiation of SkMSCs by targeting the miR-365a-3p

**Figure 3:**
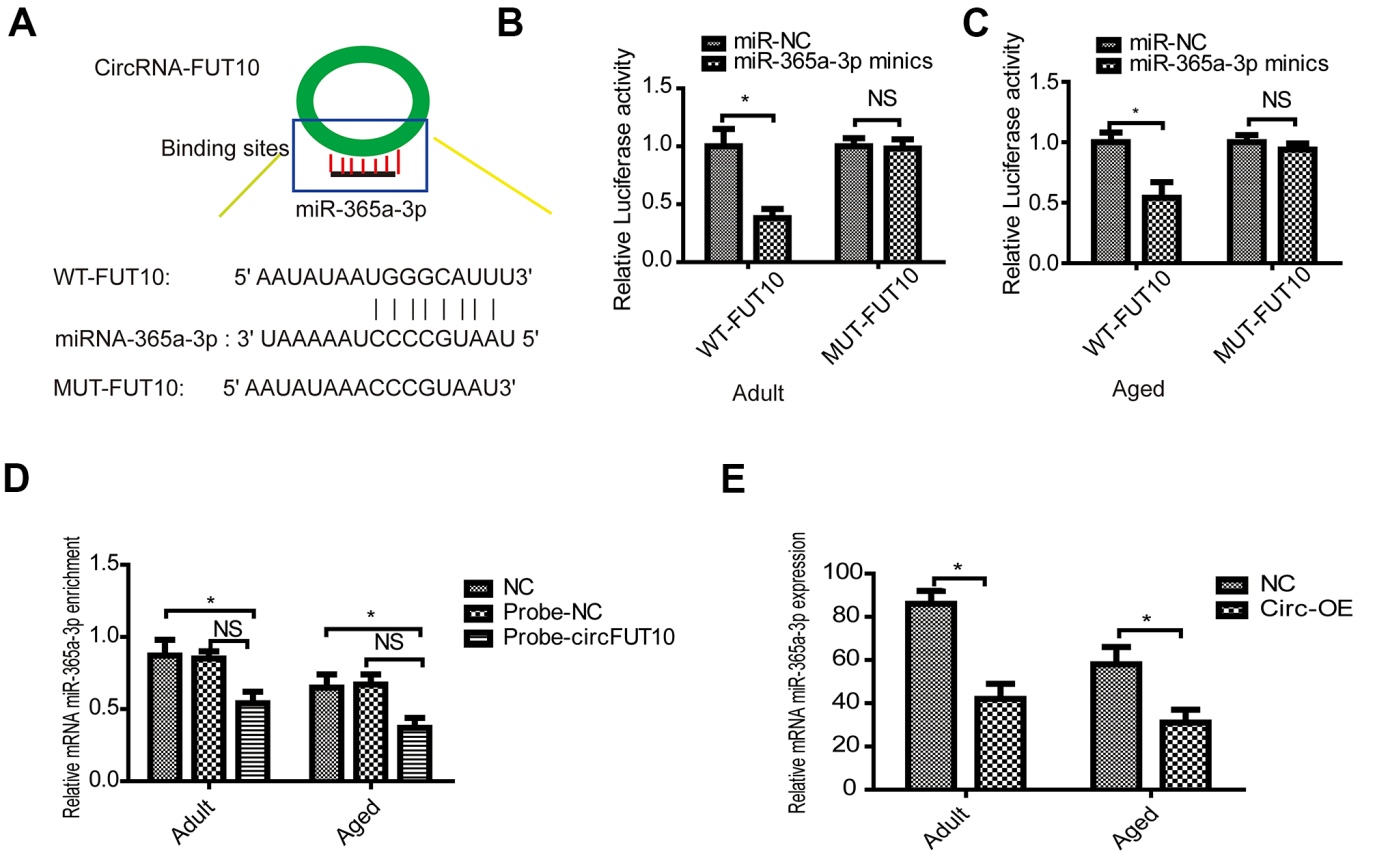
**CircRNA FUT10 sponges miR-365a-3p.** (**A**) starBase software was used to predict miR-365a-3p binding sites on circRNA FUT10. Dual-luciferase reporter assays validated the miR-365a-3p binding sites on circRNA FUT10 in (**B**) adult and (**C**) aged SkMSCs. (**D**) RNA pull-down assay results indicated that miR-365a-3p binding to circRNA FUT10 probes was enriched. (**E**) miR-365a-3p was analyzed by qPCR. Each bar represents the mean ± SEM. *P < 0.05. All experiments were performed at least three times with duplication within each individual experiment.

Previous data implied that underlying molecular mechanisms exist among circRNA FUT10, and these mechanisms were validated in our experiments. Initially, the online starBases software predicted that circRNA FUT10 can bind to the 3′ UTR regions of miR-365a-3p (Figure 3A). Furthermore, dual-luciferase reporter assay results showed that circRNA FUT10 binds to the miR-365a-3p 3′ UTR in adult (Figure 3B) and aged SkMSCs (Figure 3C). RNA pull-down assays confirmed that miR-365-3p bound circRNA FUT10 probes in SkMSCs (Figure 3D). Moreover, circRNA FUT10 overexpression in adult and aged SkMSCs led to downregulation of miR-365a-3p (Figure 3E).

**Figure 4:**
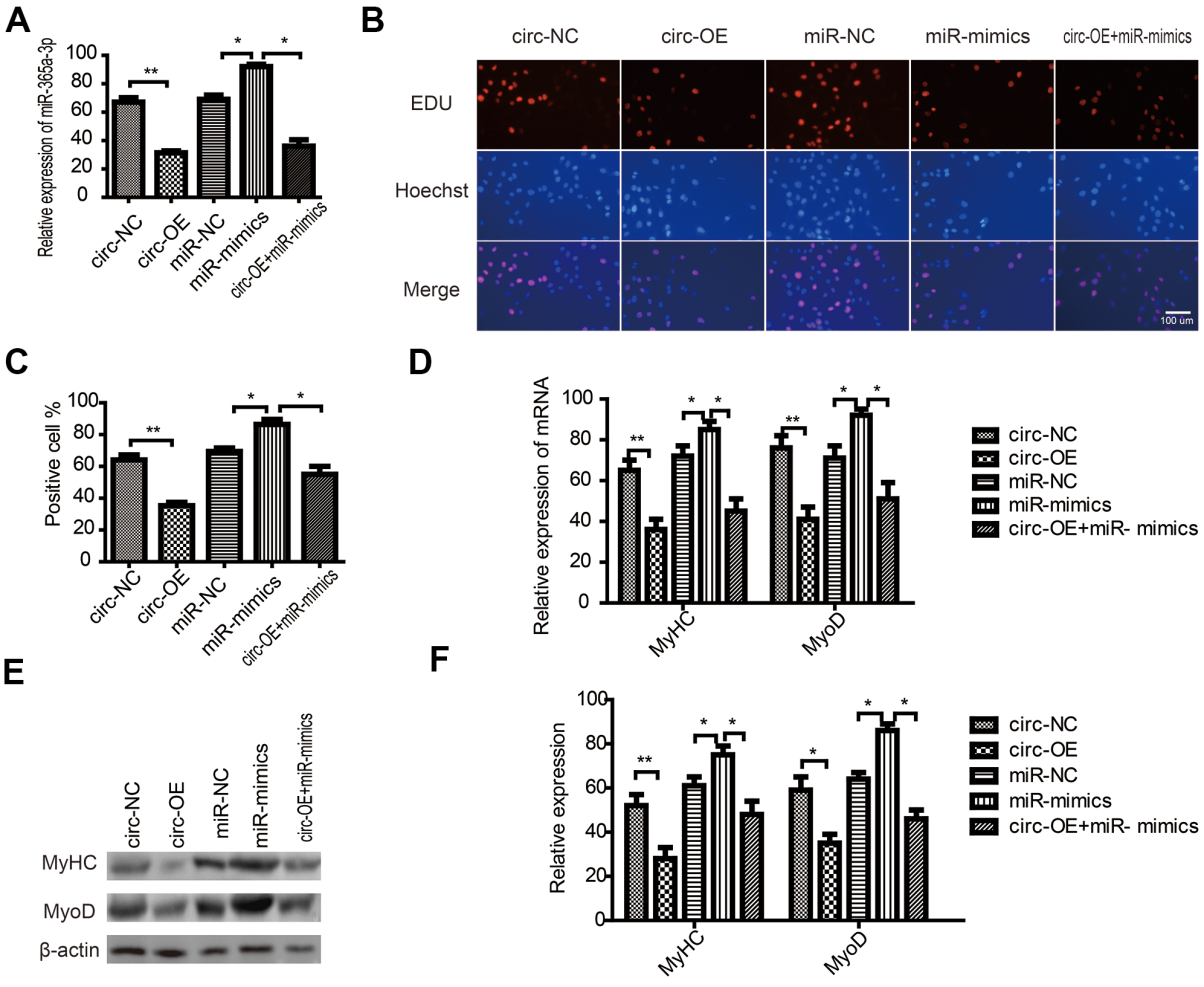
**CircRNA FUT10 suppresses SkMSC proliferation and differentiation by targeting miR-365a-3p.** (**A**) circRNA FUT10 overexpression vectors, miR-365a-3p mimics, and circRNA FUT10 + miR-365a-3p were transfected into SkMSCs. miR-365a-3p expression was examined by real-time qPCR. (**B**) EdU assay results indicated that upregulated circRNA FUT10 suppressed SkMSC proliferation by regulating miR-365a-3p. (**C**) Quantification of EdU-positive cells. (**D**) MyHC and MyoD mRNA were examined by real-time qPCR. (**E**) Western blot analysis showing MyHC and MyoD protein expression in different groups. (**F**) Quantification of MyHC and MyoD were normalized to β-actin. Each bar represents the mean ± SEM. *P < 0.05, **P < 0.01. All experiments were performed at least three times with duplication within each individual experiment.

To explore circRNA FUT10-related mechanisms in SkMSC proliferation and differentiation, we overexpressed miR-365a-3p (miR-mimic) and circRNA FUT10 in SkMSCs using overexpression vectors. qPCR analysis showed that the miR-mimic promoted miR-36a-3p expression. These effects were abrogated by circRNA FUT10 overexpression (Figure 4A). In addition, EdU assays showed that miR-365a-3p overexpression inhibited SkMSC proliferation; this effect was reversed by circRNA FUT10 overexpression (Figure 4B, 4C). Furthermore, MyoD and MyHC mRNA and protein levels were measured by qPCR (Figure 4D) and western blot (Figure 4E), respectively. The MyHC and MyoD results showed that miR-365a-3p overexpression inhibited SkMSC differentiation, while this suppression was abolished by circRNA FUT10 (Figure 4F). Thus, circRNA FUT10 suppresses SkMSC proliferation and differentiation by targeting miR-365a-3p.

### HOXA9 is the direct target of miR-365a-3p

**Figure 5:**
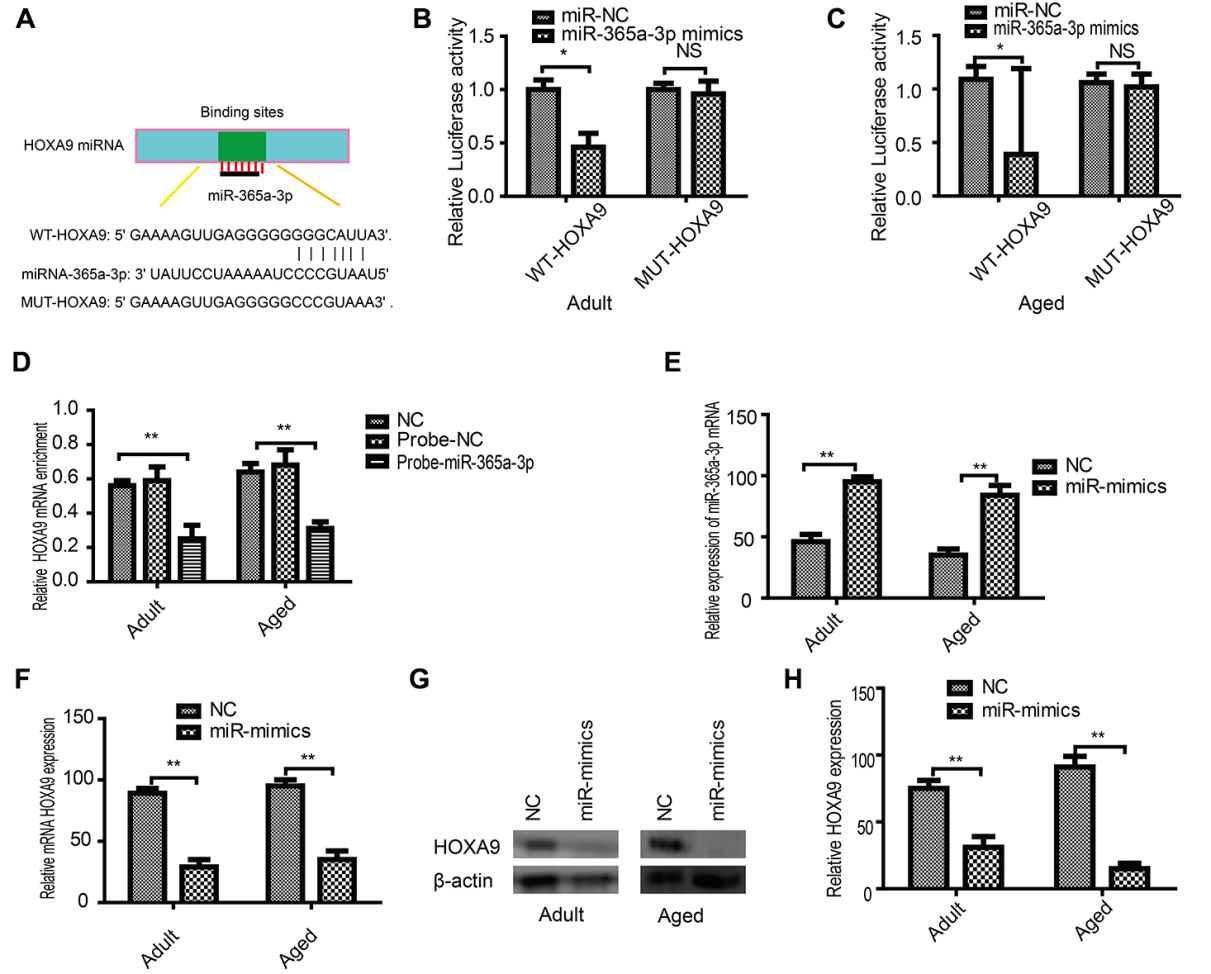
**HOXA9 is a direct miR-365a-3p target.** (**A**) Potential miR-365a-3p targets were identified in the HOXA9 3′ UTR using TargetScan software. (**B**) Adult and aged (**C**) SkMSCs were transfected with both miR-365a-3p mimic and wild-type or mutant HOXA9 3′ UTR. Binding activity was detected by dual-luciferase activity. (**D**) RNA pull-down assay results indicated that HOXA9 binding was enriched on miR-365a-3p probes. (**E**) miR-365a-3p mRNA was examined by real-time qPCR. (**F**) qPCR and (**G**) western blotting were performed to evaluate HOXA9 mRNA and protein expression in transfected cells. (**H**) Quantification of HOXA9 was normalized to β-actin. NC-antagomir, NC-mimics: control group. *P < 0.05, **P < 0.01. All experiments were performed at least three times with duplication.

Initially, *in silico* analysis predicted that miR-365a-3p can bind to the 3′ UTR of HOXA9 (Figure 5A). We used the dual-luciferase reporter gene system to verify that miR-365a-3p binds to the HOXA9 3′ UTR in adult (Figure 5B) and aged SkMSCs (Figure 5C). RNA pull-down assays confirmed that HOXA9 binding to miR-365-3p probes was enriched in SkMSCs (Figure 5D). Next, we overexpressed miR-365a-3p in adult and aged SkMSCs, which further upregulated miR-365a-3p (Figure 5E). In addition, HOXA9 mRNA and protein levels were downregulated by miR-365a-3p mimics. Thus, our results show that HOXA9 is a direct target of miR-365a-3p.

### Effect of HOXA9 on SkMSCs proliferation and differentiation

**Figure 6:**
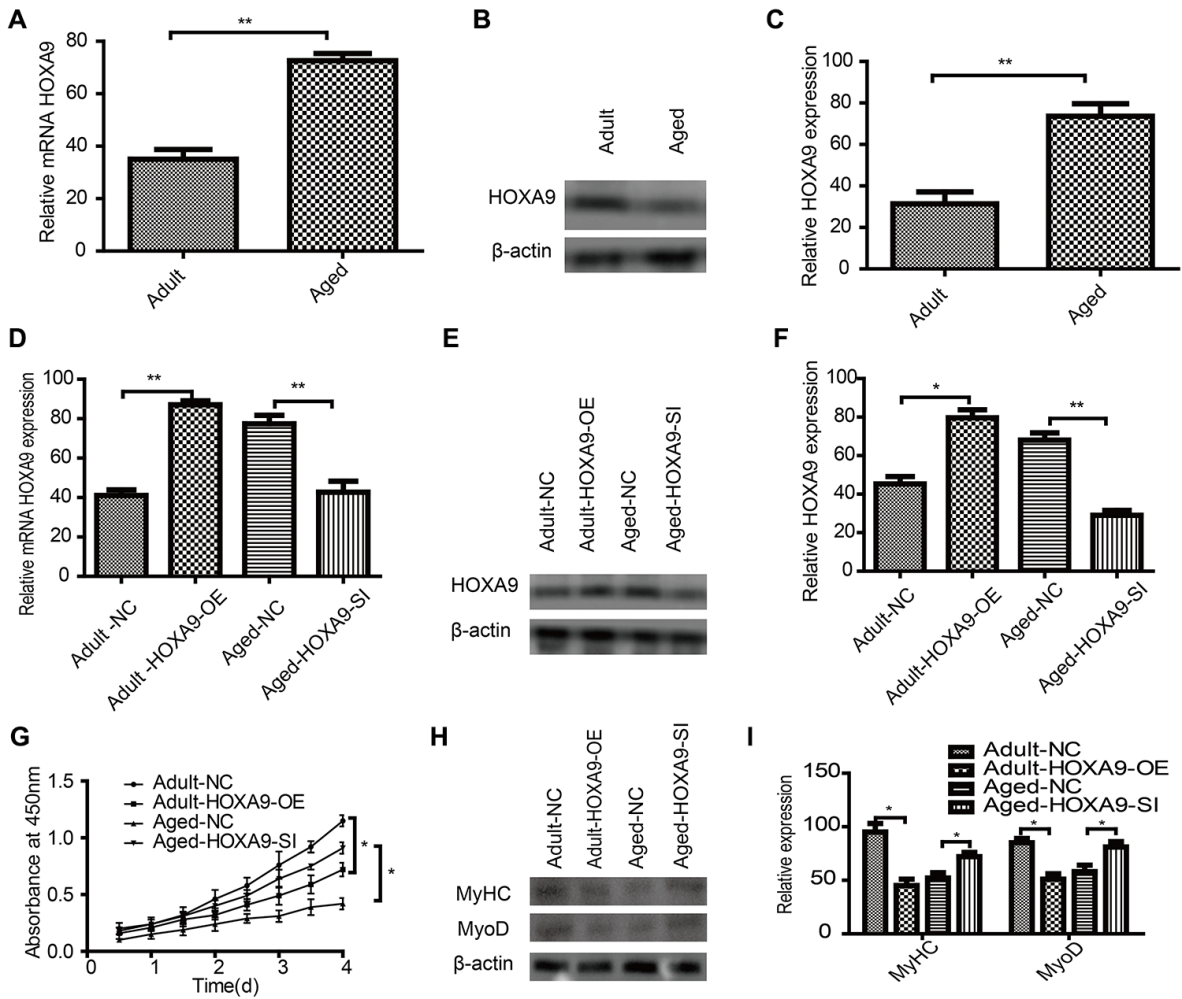
Effect of HOXA9 on proliferation and differentiation of SkMSCs (**A**) qPCR and (**B**) western blotting were performed to identify HOXA9 mRNA and protein expression in adult and aged SkMSCs. (**C**) HOXA9 was normalized to β-actin in adult and aged SkMSCs. (**D**) qPCR and (**E**) western blotting were performed to identify HOXA9 mRNA and protein expression in adult SkMSCs transfected with circRNA FUT10 overexpression vector, while aged SkMSCs were transfected with circRNA FUT10 small interfering RNA to knock down circRNA FUT10. (**F**) HOXA9 was normalized to β-actin in the above groups. (**G**) MTT assay showing the effect of HOXA9 on adult and aged SkMSC proliferation. (**H**) Western blotting was performed to identify MyHC and MyoD expression in transfected cells. (**I**) Quantification of HOXA9 was normalized to β-actin. Each bar represents the mean ± SEM. *P < 0.05, **P < 0.01. All experiments were performed at least three times with duplication within each individual experiment.

To further explore the potential function of HOXA9 in SkMSCs, HOXA9 mRNA (Figure 6A) and protein (Figure 6B, 6C) expression was evaluated in aged SkMSCs. Next, HOXA9 overexpression and knock down, respectively, caused the upregulation and downregulation of HOXA9 mRNA (Figure 6D) and protein (Figure 6E, 6F). MTT assays showed that HOXA9 overexpression inhibited adult SkMSC proliferation, while the suppression of HOXA9 promoted aged SkMSC proliferation (Figure 6G). In addition, the effect of HOXA9 on SkMSC differentiation was evaluated by western blot of MyoD and MyHC (Figure 6H). HOXA9 overexpression inhibited adult SkMSC differentiation, while the suppression of HOXA9 promoted aged SkMSC differentiation (Figure 6I). These data indicate that HOXA9 is highly expressed in aged SkMSCs, and that HOXA9 inhibits SkMSC differentiation.

### miR-365a-3p regulates the proliferation and differentiation of SkMSCs by targeting HOXA9

**Figure 7:**
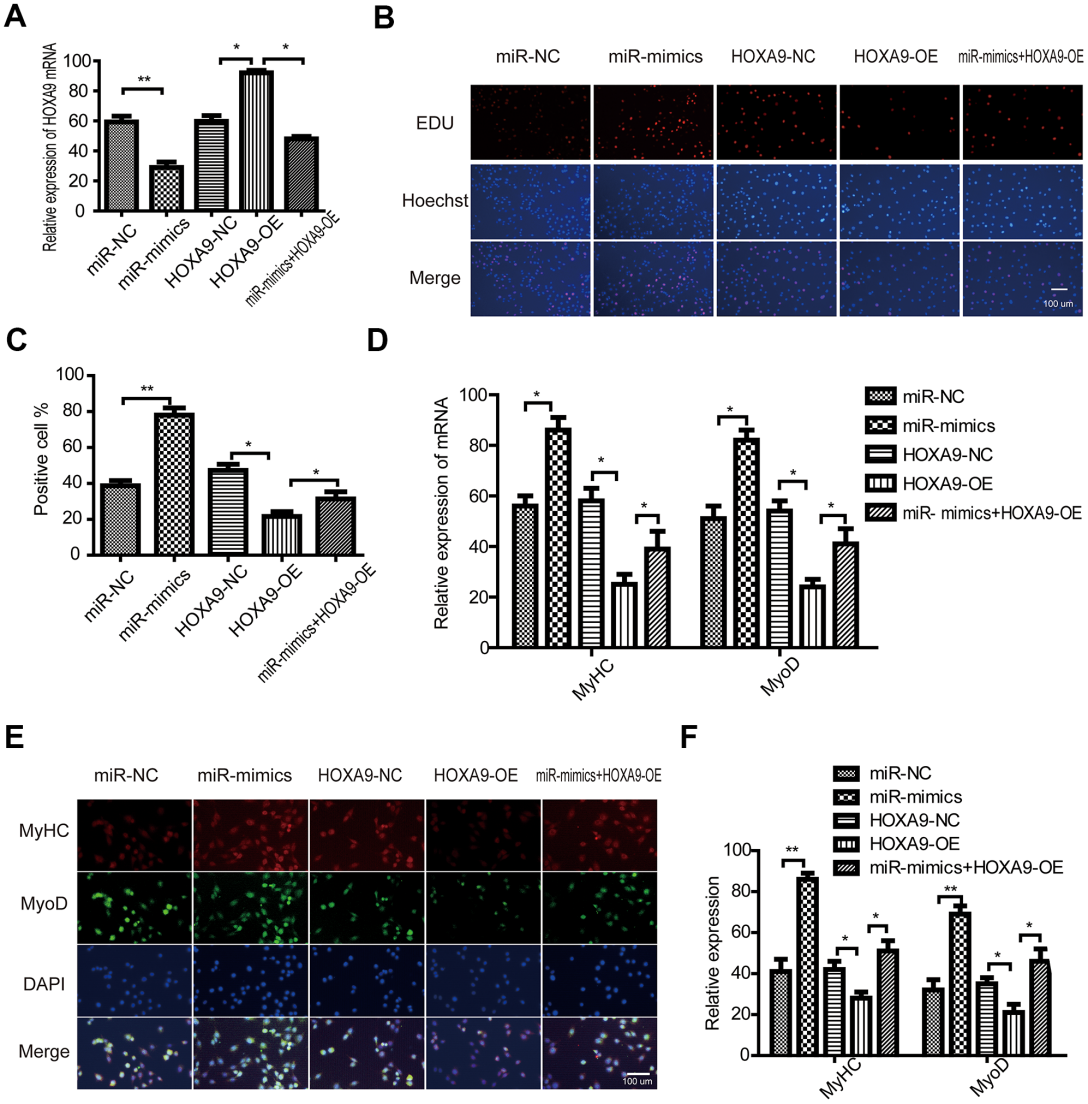
**miR-365a-3p regulates SkMSC proliferation and differentiation by targeting HOXA9.** (**A**) SkMSCs were transfected with negative control (NC) miRNA, miR-365a-3P mimic, HOXA9-NC, HOXA9 overexpression vector (HOXA9-OE), and miR-365a-3p + HOXA9-OE. HOXA9 mRNA was examined by qPCR. (**B**) SkMSC proliferation was measured by EdU assays. (**C**) Quantification of EdU-positive cells. (**D**) MyHC and MyoD mRNA were examined by real-time qPCR. (**E**) Western blot analysis showing MyHC and MyoD protein expression in different groups. (**F**) Quantification of MyHC and MyoD were normalized to β-actin. *P < 0.05, **P < 0.01. Each experiment was performed at least three times.

SkMSCs were transfected with HOXOA9 overexpression vector (HOXA9-OE) and miR-365a-3p mimic. HOXA9 was upregulated by HOXA9 vector, and this effect was abrogated by miR-365a-3p (Figure 7A). In addition, EdU assays suggested that HOXA9 overexpression suppressed SkMSC proliferation, while miR-365a-3p reversed the inhibitory HOXA9 effect (Figure 7B, 7C). Next, we observed that MyHC and MyoD mRNA expression was reduced by HOXA9 (Figure 7D), but this effect was abolished by miR-365a-3p. Immunofluorescence staining (Figure 7E, 7F) verified this result. These data confirm that miR-365a-3p regulates SkMSC proliferation and differentiation by targeting HOXA9.

### circRNA FUT10 regulated HOXA9 by sponging miR-365a-3p

**Figure 8:**
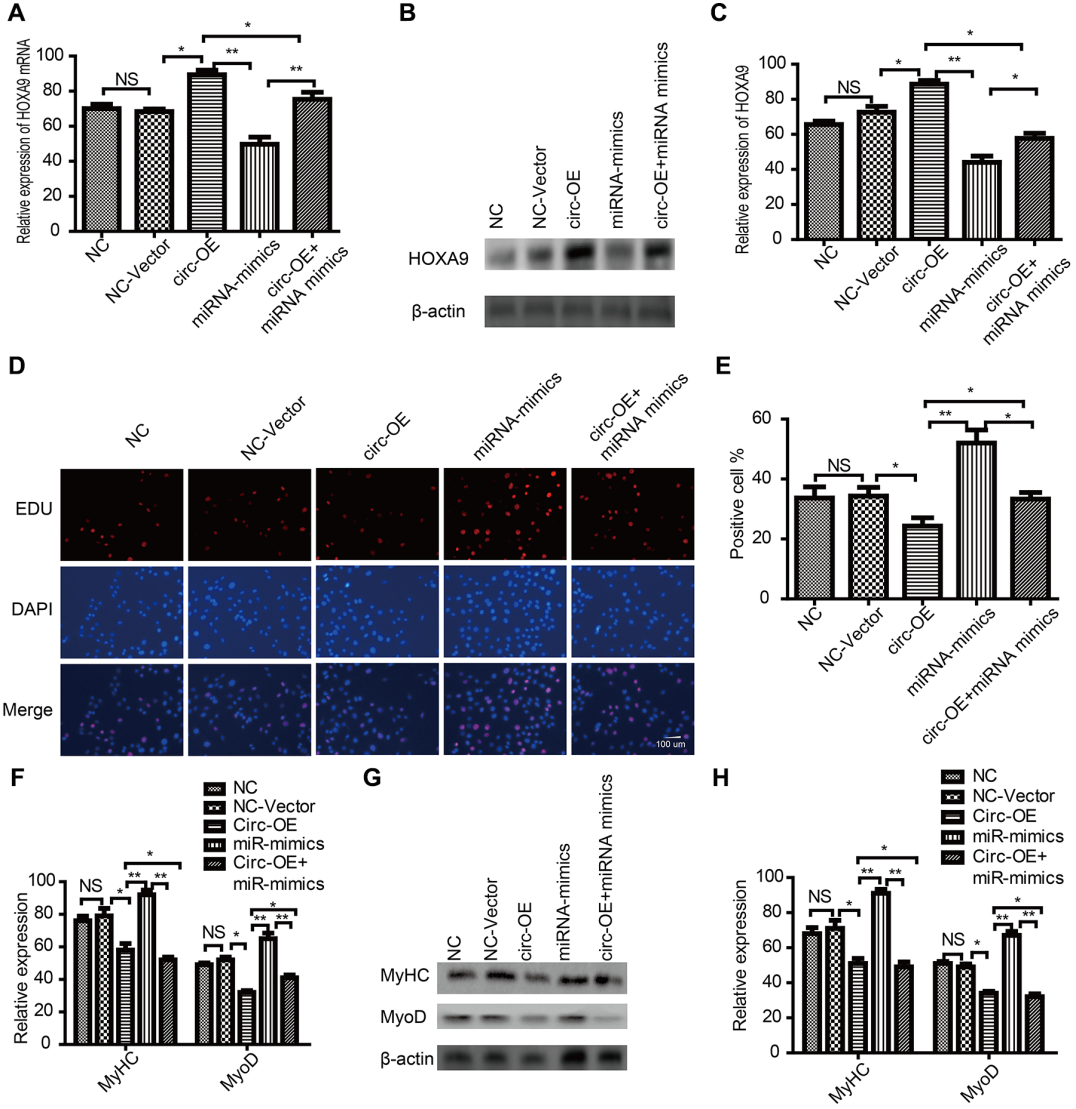
**CircRNA FUT10 regulated HOXA9 by sponging miR-365a-3p.** (**A**) SkMSCs were transfected with negative control (NC) miRNA, circRNA FUT10vector, miR-365a-3P mimic, (circ-OE), and circ-OE+miR-365a-3p. HOXA9 mRNA was examined by qPCR. (**B**) Western blot analysis showing HOXA9 protein expression in different groups. (**C**) Quantification of HOXA9 were normalized to β-actin. (**D**) SkMSC proliferation was measured by EdU assays. (**E**) Quantification of EdU-positive cells. (**F**) MyHC and MyoD mRNA were examined by real-time qPCR. (**G**) Western blot analysis showing MyHC and MyoD protein expression in different groups. (**H**) Quantification of MyHC and MyoD were normalized to β-actin. *P < 0.05, **P < 0.01. Each experiment was performed at least three times.

SkMSCs were transfected with circRNA FUT10 overexpression vector (circ-OE) and miR-365a-3p mimic. Q-PCR (Figure 8A) and western blot (Figure 8B, 8C) demonstrated that HOXA9 is upregulated by circ-OE, and this effect was abrogated by miR-365a-3p. In addition, EdU assays suggested that miR-365a-3p mimic promote SkMSC proliferation, while circRNA FUT10 weakened the promote effect (Figure 8D, 8E). Next, we observed that MyHC and MyoD mRNA expression was upregulated by miR-365a-3p (Figure 8F), but this effect was abolished by circRNAFUT10. Western blot (Figure 8G, 8H) verified this result. These data confirm that circRNA FUT10 regulated HOXA9 by sponging miR-365a-3p.

## DISCUSSION

Accumulating evidence has demonstrated that circRNAs are involved in cell aging. A better understanding of the molecular mechanism involved in the development and progression of degenerative muscle disease may force us to explore the underlying mechanisms of SkMSCs aging. In this study, we investigated the expression of circRNA FUT10 in aged SkMSCs. Furthermore, we identified its roles in SkMSC aging and showed that circRNA FUT10 binds miR365a-3p and HOXA9. We also validated that circRNA FUT10 positively regulates HOXA9 by sponging miR-365-3p in SkMSC aging.

Recently, the regulatory potential of circRNAs and their role in the development and progression of muscle diseases have been reported [15, 25, 26]. Studies have also suggested that circRNA is involved in cellular aging [27–29]. One recent study reported that circLMO7 regulates myoblast differentiation and survival via miR-378a-3p [30]. circBBS9 is suggested to play active roles in muscle aging by mediating the benefits of aerobic training intervention [31]. Interestingly, circRNA FUT10 has been reported to promote proliferation and inhibit cell differentiation [11], as well as to reduce proliferation and facilitate myoblast differentiation [12]. In our experiments, we observed that circRNA FUT10 is significantly upregulated in aged SkMSCs.

miR-365a-3p was predicted to be a downstream target of circRNA FUT10. Previously, miR-365a-5-3p was identified to be involved in cell proliferation [32–34]. We hypothesized that miR-365a-3p sponges circRNA FUT10 to inhibit SkMSC aging. In our study, miR-365a-3p overexpression promoted proliferation and differentiation, and eliminated the effect of circRNA FUT10. A direct interaction between miR-365a-3p and circRNA FUT10 was also confirmed, suggesting that miR-365a-3p is negatively regulated by circRNA FUT10. Our results suggest that miR-365a-3p can be negatively regulated by circRNA FUT10. Similarly, this argument was validated by some researches [35–37].

Furthermore, we used TargetScan analysis to predict targets with miR-365a-3p binding sites. Among the candidates, HOXA9 was verified as a downstream target of miR-365a-3p by luciferase reporter assays. Several studies report that HOXA9 is involved in cell proliferation [38–40]. One study demonstrated that HOXA9 may promote denervated muscle atrophy by strongly upregulating MLL1 and WDR5 expression and inhibiting myogenic differentiation [23]. Furthermore, HOXA9 deletion improves satellite cell function and muscle regeneration in aged mice, whereas HOXA9 overexpression mimics age-associated defects in satellite cells from young mice, which can be rescued by inhibiting HOXA9-targeted developmental pathways. Clearly, HOXA9 is related to satellite cell aging. Our results demonstrate that HOXA9 is overexpressed in aged SkMSCs compared with control samples and that miR-365a-3p reverses the suppressive effect of HOXA9 on SkMSC proliferation and differentiation. In accordance with these observations, some studies reported that HOXA9 is the direct target of miR-365a-3p [41–43]. Furthermore, circRNA FUT10 downregulation suppressed SkMSC proliferation and viability. These effects were reversed by miR-365a-3p overexpression and HOXA9 silencing. Finally, we examined the percentage of Pax7+ cells derived from primary cultures of different muscle tissues. Our results indicate that the circRNA FUT10/miR-365a-3p/HOXA9 axis regulates SkMSC aging.

In summary, we observed that circRNA FUT10 is highly expressed in aged SkMSCs and suppresses cell proliferation and differentiation. circRNA FUT10 competitively binds miR-365a-3p to attenuate the suppressive effect of miR-365a-3p on HOXA9. These findings provide insights into SkMSC aging and provide a potential target for the treatment of degenerative muscle disease.

## MATERIALS AND METHODS

### Cell culture and transfection

Isolated single myofiber-associated cells were prepared using limb muscles obtained from 2-week-old and 6-month-old female BALB/c mice (30-200 g, maintained in a 12:12 h light/dark cycle at 23° C and 50-70% humidity). Animal experiments were approved by The Institutional Animal Care and Use Committee at The Third Affiliated Hospital of Southern Medical University (Guangzhou, China). All animals were purchased from The Guangdong Medical Laboratory Animal Center (Guangzhou, China). SkMSCs were dissected from mice tibialis muscles using enzymatic dissociation (0.2% collagenase, Gibco;CA, USA) at 37° C for 60 min. Following be filtered by a 80-μm filter (Bioss, Beijing, China), cells were stained for the isolation of particular cell populations by flow cytometry and fluorescence-activated cell sorting (FACS). Then, cells were cultured in DMEM (Gibco; Thermo Fisher Scientific, Inc.) supplemented with 20% FBS (HyClone; GE Healthcare life Sciences) and 1% chick embryo extract (Gemini Bio Products). Cells differentiation was induced by DMEM with 2% heat-inactivated horse serum (Gibco; Thermo Fisher Scientific, Inc.). The overexpression vectors (Roche, Switzerland) for circRNA FUT10 (circ-OE) and HOXA9 (HOXA9-OE), miR-365a-3p mimic and inhibitor (Sigma-Aldrich; Merck KGaA) and the short harpin RNA (shRNA) (Bioss, Beijing, China) for circRNA FUT10 (circ-SI) and small interfering RNA (siRNA) (Invitrogen, CA, USA) for HOXA9 (HOXA9-SI) were pretransfected into SkMSCs, respectively. After cells were plated for six hours, the vectors were added. The above vectors were delivered into SkMSCs by using a Lipofectamine 2000 reagent (Invitrogen, CA, USA) according to the manufacturer’s instructions. RNA can be extracted after 24 hours, and the protein can be extracted after 48 hours.

### RNA isolation and qRT-PCR amplification

Total RNA was extracted by TRIzol® reagent (Thermo). Following cDNA be obtained by the SuperScript® cDNA Synthesis Kit (Thermo), it was transferred into a 20-μl PCR mixture with 2× UltraSYBR Mixture (Gibco;CA, USA). The primers were as follows: circRNA FUT10 forward: 5′-GCGGATGCTTGCTTCTTC-3′ and reverse, 5′-GAGGCTCTGCTTCCATTTGT-3′); miR-365a-3p forward 5′- ACACTCCAGCTGGGTCCGAGCCTGGGTCTC-3′ and reverse 5′-TGGTGTCGTGGAGTCG-3′; U6 forward: 5′--CTCGCTTCGGCAGCACA-3′ and reverse: 5′-AACGCTTCACGAATTTGCGT-3′; HOXA9 forward: 5′-CTTACCCAAGCTTCACTCACC-3′ and reverse: 5′- AAGAGGCCTGGTGCTACTAC-3′; β-actin forward: 5′-TCATGAAGTGTGACGTGGACATC-3′ and reverse: 5′-CAGGAGGAGCAATGATCTTGATCT-3′. U6 or β-actin was used as an internal control to normalize target gene transcripts.

### Luciferase reporter assay

The amplified miR-365a-3p inhibitor sequence and miR-NC (Thermo Fisher Scientific, Inc) were transfected into SkMSCs using Lipofectamine® 2000 (Invitrogen; Thermo Fisher Scientific, Inc.) according to the manufacturer's instructions. To identify the binding sequences and uniform resource locator, a luciferase reporter assay was used. Then, miR-365a-3p inhibitor or miR-NC and the pRL-TK vector (Promega Corporation) carrying the mutant (mut) or wild-type (wt) circRNA FUT10 and HOXA9 3' untranslated region (3'-UTR) were co-transfected into SkMSCs using Lipofectamine® 2000 (Invitrogen; Thermo Fisher Scientific, Inc.). Three days later, cells were lysed with Dual-Glo®Reagent (Promega Corporation), and luciferase activity was measured using a Dual-Luciferase Reporter Assay System (Promega Corporation).

### RNA pull-down assay

RNA pull-down assays were performed to identify the binding sites of miR-365a-3p with circRNA FUT10 and the 3′ UTR regions of HOXA9 mRNA. The biotin-labeled circRNA FUT10 and HOXA9 probes (Invitrogen, CA, USA) were designed and constructed. Then, the above probe-streptavidin Dynabeads were incubated with SkMSCs at 37° C for 12 hours. Finally, real time qPCR was performed to assess miR-365a-3p enrichment according to the manufacturer's instructions.

### Western blot analysis

Proteins extracted from cell lysates or supernatant culture medium were subjected to electrophoresis. After proteins were transferred and blocked, strips were subjected to rabbit anti-β-actin (1:1000; ab8227, Abcam, Cambridge, UK), anti-HOXA9 (1:1000; EPR3655(2) ab140631), anti-MyHC (1:1000; ab207926), anti-MyoD (1:1000; ab133627) antibodies overnight at 4° C.

### 3-(4,5-dimethylthiazol-2-yl)-2,5-diphenyltetrazolium bromide (MTT) assay

An MTT assay was conducted to evaluate the role of circRNA FUT10 in SkMSCs. SkMSCs stably transfected with NC or HOXA9-SI or HOXA9-OE were seeded into 96-well plates at a density of 4×10^4^ cells/well and incubated for 24 hours. Then, 20 μL of MTT solution (5 mg/ml) was added into each well and incubated for an additional 4 hours. After adding 160 μL/well of DMSO to each well, the optical density (OD) value of each well was measured at 450 nm by a spectrophotometer (Philips, China).

### 5-Ethynyl-2′deoxyuridine (EdU) assay

Cells were seeded at a density of 8×10^4^ cells/well in 6-well plates and cultured for 24 h at 37° C. then, cells were treated with 10 μM EdU working solution growth medium for 2 h in the dark. Then, the cells were treated with 4% paraformaldehyde for 20 min, followed by 2 mg/ml glycine and 0.5% Triton X-100 for 15-20 min at room temperature. After incubated with Hoechst 33342 for about half an hour, cells were subjected to an Axiovision 4.8 camera attached to an Axio Observer Z1 inverted microscope (Carl Zeiss, Inc.).

### Immunohistochemistry (IHC)

Tissues were fixed in 10% neutral-buffered formalin and then embedded in paraffin. Paraffin-embedded tissues were deparaffinized and rehydrated for further 3,3'-diaminobenzidine peroxidase (DAB) immunohistochemical staining. Then, after proteolytic digestion and peroxidase blocking, slides were incubated with antibodies against Ki67 (1:100, ab15580, Abcam, USA) overnight at 4° C.

### Flow cytometry assay

To assess stem phenotype conversion, cells were resuspended in 200 μL PBS and incubated with anti-Pax7-BV510 (4 μl/ ml) at 4° C for 1 hour. After the cells were washed three times with PBS and suspended in 100 μL PBS, the analysis was performed on a Flow CytoFLEX (Beckman Coulter, USA).

### Statistical analysis

Statistical significance was determined by performing Student's t-test for comparisons between two groups and one-way analysis of variance followed by NDTukey's post hoc test for comparisons between more than two groups using the GraphPad 5.0 (GraphPad Software, La Jolla, CA). Unless otherwise stated all data are expressed as the mean ± SEM. A difference was considered statistically significant at a level of P < 0.05.

### Availability of data and materials

The datasets used and/or analyzed during the current study are available from the corresponding author on reasonable request.

### Ethics approval and consent to participate

The study was approved by the Ethics Review Committee of The Third Affiliated Hospital of Southern Medical University (Guangzhou, China). The animal study followed the Guidelines for the Animal Care and Use approved by The Third Affiliated Hospital of Southern Medical University.
